# A phase I study of LY3164530, a bispecific antibody targeting MET and EGFR, in patients with advanced or metastatic cancer

**DOI:** 10.1007/s00280-018-3623-7

**Published:** 2018-06-20

**Authors:** Amita Patnaik, Michael Gordon, Frank Tsai, Kyri Papadopoulous, Drew Rasco, S. Muralidhar Beeram, Siqing Fu, Filip Janku, Scott M. Hynes, Sushma R. Gundala, Melinda D. Willard, Wei Zhang, Aimee Bence Lin, David Hong

**Affiliations:** 1 South Texas Accelerated Research Therapeutics (START), 4383 Medical Drive, Suite 4026, San Antonio, TX USA; 2 HonorHealth Research Institute, Scottsdale, AZ USA; 3 Cancer Medicine Division, MD Anderson Cancer Center, The University of Texas, Houston, TX USA; 4 Eli Lilly and Company, Indianapolis, IN USA

**Keywords:** Phase I, MET, EGFR, Bispecific

## Abstract

**Purpose:**

The phase I study characterized the safety, pharmacokinetics, anti-tumor activity, and recommended phase II dose/schedule of LY3164530 in patients with advanced or metastatic cancer.

**Methods:**

Patients received LY3164530 on days 1 and 15 (Schedule 1: 300, 600, 1000, and 1250 mg) or Days 1, 8, 15, and 22 (Schedule 2: 500 and 600 mg) of each 28 days cycle. Dose escalation used a modified toxicity probability interval model.

**Results:**

Dose escalation defined a maximum tolerated dose (MTD) of 1000 mg on Schedule 1 and 500 mg on Schedule 2. Treatment-emergent adverse events related to study treatment were consistent with epidermal growth factor receptor (EGFR) inhibition and included maculopapular rash/dermatitis acneiform (83%, Grade 3/4 17%), hypomagnesemia (55%, Grade 3/4 7%), paronychia (35%), fatigue (28%, Grade 3/4 3%), skin fissures (24%), and hypokalemia (21%, Grade 3/4 7%). Partial response was achieved in three patients on Schedule 2 with colorectal cancer (*n* = 2) or squamous cell cancer. Overall response rate (ORR) was 10.3%, disease control rate (ORR + stable disease [SD]) was 51.7 and 17.2% of patients had SD ≥ 4 months. The in vivo stability of the bispecific antibody was confirmed. Schedule 2 provided greater and more consistent inhibition of mesenchymal-epithelial transition (MET)/EGFR throughout the dosing interval than Schedule 1.

**Conclusions:**

Although this study defined the LY3164530 MTD and pharmacokinetics on both schedules, significant toxicities associated with EGFR inhibition and lack of a potential predictive biomarker limit future development. Nonetheless, the results provide insight into the development of bispecific antibody therapy.

**Electronic supplementary material:**

The online version of this article (10.1007/s00280-018-3623-7) contains supplementary material, which is available to authorized users.

## Introduction

Epidermal growth factor receptor (EGFR) and the mesenchymal-epithelial transition factor (MET; also known as hepatocyte growth factor [HGF] receptor) are receptor tyrosine kinases that are coexpressed in many tumors, including non-small cell lung cancers (NSCLC), colorectal cancers (CRC), and head and neck cancers (HNSCC) [1–3]. EGFR signaling plays a crucial role in tumor biology by modulating cellular proliferation, angiogenesis, metastasis, and survival of cancer cells. Dysregulation of this pathway has been implicated in tumorigenesis [4]. Aberrant MET signaling resulting from the overexpression of MET, activating mutations in *MET*, transactivation, autocrine or paracrine signaling, or *MET* gene amplification, have also been implicated in the development/progression of many human cancers [5, 6]. Through the investigation of cancer therapy outcomes, an important relationship between EGFR and MET signaling was established. MET is a critical player in developing resistance to targeted therapies, including therapies directed at EGFR [7]. Similarly, mutations in *EGFR* and downstream genes such as *KRAS*, histologic transformation, and the activation of alternative pathways, including the MET signaling pathway, have been identified as mechanisms of resistance to EGFR-targeted therapies [8, 9]. Consequently, blocking one receptor tends to upregulate the other, leading to resistance to single-agent treatment [10]. Amplification of MET and/or high levels of HGF ligand expression have been observed in NSCLC patients with intrinsic or acquired resistance to tyrosine kinase inhibitors of EGFR, including erlotinib and gefitinib [10, 11]. Conversely, MET-amplified lung cancer cells exposed to MET-inhibiting agents for a prolonged period develop resistance via the EGFR pathway [9].

Because crosstalk between the signaling pathways controlled by these receptors has emerged as a mechanism of both cancer progression and resistance to therapy [12], dual inhibition of these targets may lead to improved outcomes for patients with MET- and EGFR-driven cancers. In addition, simultaneous inhibition may overcome or delay therapeutic resistance compared to the blockade of just one pathway. The dual inhibition of MET and EGFR has been explored using a combination of separate MET and EGFR inhibitors [13, 14].

LY3164530 is an engineered bispecific antibody designed to neutralize, internalize, degrade, and disrupt signaling via both the MET and EGFR receptors. The antibody consists of two identical heavy chains and two identical light chains, with an immunoglobulin G4 antibody to MET (emibetuzumab, LY2875358 [14]) and a single-chain variable fragment to EGFR fused to the N-terminus of each heavy chain. The bispecific antibody has increased avidity in cells expressing both receptors [15] and is active in ligand-dependent and independent models. In cells expressing high MET and EGFR, LY2875358 is superior in internalizing/degrading EGFR (wild-type and mutant forms) over a combination of emibetuzumab and cetuximab (an approved EGFR inhibitor [16]); similarly, in comparison with the combination of individual antibodies, LY3164530 leads to greater anti-tumor activity in in vivo models and has the ability to better overcome HGF-mediated resistance to erlotinib, gefitinib, lapatanib, or vemurafenib in in vitro assays [17]. Emibetuzumab and cetuximab are both well-tolerated in patients with cancer [14, 16]. Therefore, the safety profile of LY3164530 was expected to be consistent with these compounds.

Study I7H-MC-JNBA was a phase I study to characterize the safety and determine the recommended phase II dose and schedule of LY3164530 in patients with advanced or metastatic cancer. A literature search revealed that this is the first report of clinical data with a bispecific antibody targeting both MET and EGFR in a single molecule. Although the future development of this agent is limited due to the marked EGFR-associated toxicities and lack of a clear predictive biomarker, these results provide key insights into how bispecific antibody therapies can be improved and utilized in future trials.

## Materials and methods

### Study design

This was a multicenter, non-randomized, open-label, phase I dose escalation study of LY3164530 in patients with advanced or metastatic cancer. The study was conducted at three centers in the United States between August 2014 and March 2017. Patients (*N* = 29) were treated with LY3164530 intravenously on Days 1 and 15 (Schedule 1, every 2 weeks [Q2W]: 300, 600, 1000, and 1250 mg) or on Days 1, 8, 15, and 22 (Schedule 2, weekly [QW]: 500 and 600 mg) of each 28-day cycle. The planned treatment period concluded when disease progression or unacceptable toxicity occurred. Tumor restaging evaluations were performed every two cycles (8 weeks). Patients were monitored for 28 days following discontinuation of study treatment. Those patients who received benefit from the study drug could be treated until one or more of the discontinuation criteria were fulfilled.

### Patient eligibility

The study included patients who had advanced and/or metastatic cancer refractory to standard therapies. Patients were also permitted to participate if they were not eligible for standard curative therapy or refused standard therapies. In particular, patients were included in the study if they met the following inclusion criteria during screening: ≥ 18 years old with histological or cytological evidence of advanced/metastatic cancer; Eastern Cooperative Oncology Group performance score (ECOG PS) of 0–1; presence of measurable and/or non-measurable disease as defined by Response Evaluation Criteria in Solid Tumor guidelines (RECIST) v1.1; adequate hematologic, hepatic, and renal organ function; recovered from the acute effects of prior therapy, radiation treatment, and surgery; and use of reliable method of birth control during the study and a negative pregnancy test for 3 months following the last dose of study drug for females of child-bearing potential.

Patients were not included in the study if any of the following exclusion criteria were met: serious pre-existing medical or cardiac condition; active central nervous system or brain metastases; second primary malignancy (including acute or chronic leukemia); active infection; corrected QTc > 470 msec; known allergy/hypersensitivity to any of the study drug components; and receipt of another investigational product within 28 days of the start of treatment. The study was conducted according to the principles of good clinical practice, applicable laws and regulations, and the 1964 Declaration of Helsinki and its later amendments or comparable ethical standards. Each institution’s review board approved the study. All patients signed an informed consent document before study participation.

### Objectives

The primary objective of the study was to determine the recommended phase II dose and schedule of LY3164530. Secondary objectives included safety and toxicity, PK, and anti-tumor activity of LY3164530. Other exploratory objectives included biomarker assessment via immunohistochemistry (IHC) and mutational analysis for association with tumor response and/or safety.

### Dose escalation

Dose escalation was conducted using a modified toxicity probability interval model (mTPI) [18]. In the mTPI, the number of patients per cohort was not fixed (variable 3–20 patients), but a minimum of three patients was required for each dose level. Dose limiting toxicity (DLT) was defined as an adverse event (AE) during Cycle 1 that was considered by the investigator to be at least possibly related to LY3164530 and fulfilled any of the following criteria: (a) Grade ≥ 3 non-hematological toxicity, with the exception of nausea, vomiting, diarrhea, and constipation controllable with treatment (Grade 3 or 4 nausea, vomiting, or diarrhea were considered DLTs if they persisted for more than 48 h despite supportive intervention); Grade 3 rash that resolved with treatment to ≤ Grade 1 within 14 days; Grade 3 or 4 asymptomatic electrolyte abnormalities that responded to standard treatment (Grade 3 elevations of alanine aminotransferase and/or aspartate aminotransferase lasting fewer than 8 days, without the evidence of other hepatic injury, in the setting of pre-existing hepatic metastasis and baseline elevation of these values, were not considered a DLT if agreed by the study investigator and Eli Lilly and Company clinical research scientist); (b) Grade 4 neutropenia or leukopenia of > 7 days duration; (c) Grade 3 thrombocytopenia with bleeding or Grade 4 thrombocytopenia of any duration; (d) any febrile neutropenia; and (e) any other significant toxicity deemed by the primary investigator and Eli Lilly and Company clinical research personnel to be dose limiting. A DLT-equivalent toxicity was defined as an AE occurring in Cycle 2 and beyond that would have met the criteria for a DLT had it occurred in Cycle 1. The study was designed to identify a dose level with a dose-limiting target toxicity rate of 30%. However, since the exact target toxicity rate is almost never achieved for a dose, the mTPI method considers an equivalence interval (EI) around the target toxicity rate. For this study, the EI was calibrated to be 28.7, 30.1% and this drove the mTPI escalation, de-escalation, and stay rules that defined the maximum tolerated dose (MTD).

### Pharmacokinetic analysis

Pharmacokinetic analyses were conducted on patients who had received at least 1 dose of study drug and had blood samples collected for the measurement of serum concentrations of LY3164530. Concentration–time profiles of LY3164530 in the serum samples were analyzed by MET- and EGFR-specific enzyme-linked immunosorbent assays (validated at Charles River Laboratories located in Senneville, Quebec, Canada). The PK parameters for analysis included maximum serum concentration (*C*_max_), minimum serum concentration (*C*_min_), time of maximum serum concentration (*t*_max_), systemic clearance (CL), volume of distribution at steady state (*V*_ss_), elimination half-life (*t*_1/2_), area under the serum concentration versus time curve over the dosing interval (AUC_0–τ_), and the average serum concentration over the dosing interval (*C*_av,τ_) for LY3164530. The ratio of AUC_0–τ_ (Cycle 1, Day 15 or Day 22)/AUC_0–τ_ (Cycle 1, Day 1) was reported as the intracycle accumulation ratio, and the ratio of AUC_0–τ_ (Cycle 2, Day 1)/AUC_0–τ_ (Cycle 1, Day 1) was reported as the intercycle accumulation ratio for each schedule of administration. All PK parameters were computed by standard non-compartmental methods of analysis using Phoenix WinNonlin® Enterprise Version 6.4 (Certara Corporation®) on a computer that met the minimum system requirements for this program.

### Safety assessment

All patients exposed to study drug were evaluated for safety and toxicity. AEs were collected, and the severity was graded as per the National Cancer Institute—Common Terminology Criteria for Adverse Events Version 4.03. The investigators determined the treatment-emergent AE (TEAE) relatedness to study drug and severity. Exposure to study drug, including dose modifications, and the duration of treatment were evaluated. Electrocardiograms, clinical laboratory tests, and vital signs were also monitored. Samples were collected to detect the presence of anti-drug antibodies (ADA).

### Efficacy assessment

Radiological tumor assessments were performed at baseline and evaluated every two cycles for the rate of tumor growth using objective measures (e.g., computed tomography or magnetic resonance imaging scan) by RECIST v. 1.1. Best overall response was summarized as complete response (CR), partial response (PR), stable disease (SD), or progressive disease (PD). The overall response rate was calculated as the best overall response of CR or PR. The disease control rate was calculated as the proportion of patients with best overall response of CR, PR, or SD (for ≥ 2 months).

### Biomarker analysis

All patients underwent a pre-treatment tumor biopsy to determine MET and EGFR expression and amplification. Tumor tissue was stained for IHC of MET and EGFR. Membrane MET and EGFR staining intensity was determined for each cell in a fixed field. An H-score was then assigned using the formula: 1 × (% cells 1+) + 2 × (% cells 2+) + 3 × (% cells 3+), and the percent of cells staining at a 3 + intensity was calculated. IHC results were analyzed by best overall response. MET and EGFR amplification were determined by ratio and copy number via fluorescent in situ hybridization (FISH). *KRAS* and *EGFR* somatic mutations were also assessed using Therascreen® KRAS and EGFR Rotor-Gene Q PCR assays.

## Results

### Patient disposition

A total of 36 patients were screened for eligibility, and 29 patients were enrolled in the study and received at least 1 dose of study drug. Based on the mTPI escalation rules, patients were assigned to the following doses for Schedule 1: 300 mg (*n* = 3), 600 mg (*n* = 3), 1000 mg (*n* = 11), and 1250 mg (*n* = 3); and for Schedule 2: 500 mg (*n* = 5) and 600 mg (*n* = 4). Primary reasons for discontinuation from the study included PD (*n* = 20), withdrawal by subject (*n* = 5), physician decision (*n* = 2), and AEs (*n* = 2).

### Patient characteristics

**Fig. 1:**
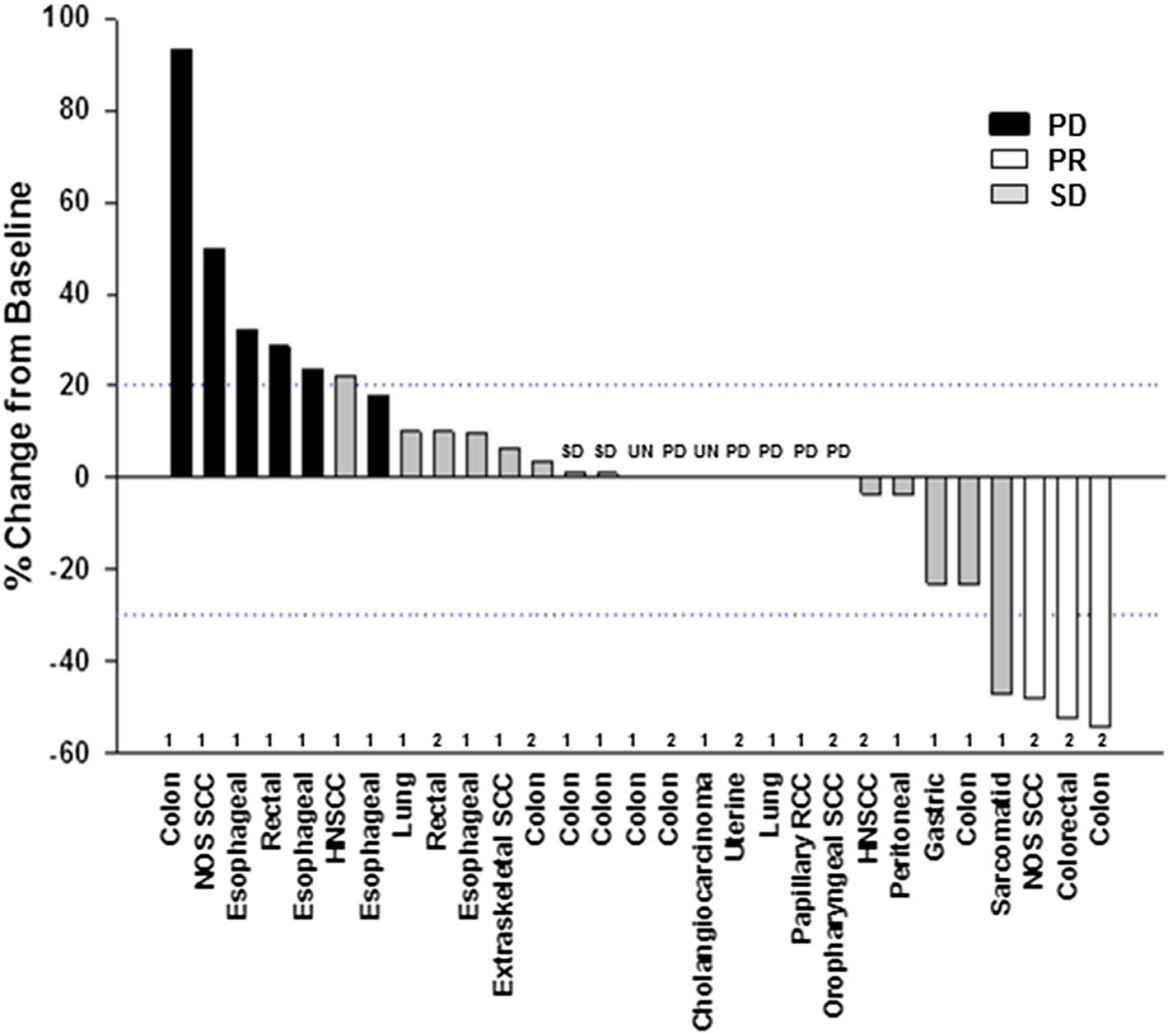
Percent change from baseline in tumor growth with schedule allocation (1 or 2), tumor type and efficacy outcome. *HNSCC* head and neck cancers, *NOS SCC* not otherwise specified squamous cell carcinoma, *PD* progressive disease, *PR* partial response, *RCC* renal cell carcinoma, *SCC* squamous cell carcinoma, *SD* stable disease, *UN* unknown

With the exception of gender distribution, the demographic and baseline characteristics of the patients were similar between Schedules 1 and 2. A total of 35.0% of the patients in Schedule 1 were female, while 55.6% of the patients treated on Schedule 2 were female. For the overall population, the median age was 60 years (range 38–76 years), median weight was 77.1 kg (range 46.3–119.7 kg), 96.6% of patients were Caucasian, and the majority of patients had an ECOG PS ≥ 1 (62.1%). The study enrolled patients with many different advanced cancers, including colon (Schedule 1 *n* = 5; Schedule 2 *n* = 3), esophageal adenocarcinoma (Schedule 1 *n* = 4), rectal adenocarcinoma, not otherwise specific squamous cell carcinoma (NOS SCC), and HNSCC (Schedule 1 *n* = 1; Schedule 2 *n* = 1 for each) (Fig. 1).

### Treatment exposure and dose modifications

Median durations of treatment for patients on Schedule 1, Schedule 2, and the total population were 56.5 days (range 16–220 days), 93 days (range 37–303 days), and 72 days (range 16–303 days), respectively. The longest duration on therapy was ten cycles in a patient with NOS SCC who received 600 mg on Schedule 2. Dose modifications were common: only 55.2% of patients were administered study drug as expected. A total of 7 (24.1%) patients had a dose reduction, 10 (34.5%) patients had study drug omitted, and 15 (51.7%) patients had a delay in study drug. Notably, the most common reasons for dose reductions were AEs (3 AEs/3 dose reductions [100.0%] Schedule 1, 5 AEs/6 dose reductions [83.3%] Schedule 2); similarly, the most common reason for dose omissions were AEs (6 AEs/6 dose omissions [100.0%] Schedule 1, 12 AEs/14 dose omissions [85.7%] Schedule 2); and the most common reasons for dose delays were AEs (5 AEs/12 dose delays [41.7%] Schedule 1, 6 AEs/9 dose delays [66.7%] Schedule 2) and scheduling conflicts (7 conflicts/12 dose delays [58.3%] Schedule 1, 3 conflicts/9 dose delays [33.3%] Schedule 2). Dose reductions were most common in Cycle 2 (10.3%), but also occurred in Cycles 1 and 3–9 of treatment.

### Dose escalation, dose limiting toxicities, and maximum tolerated dose

A total of 11 patients were treated at 1000 mg as per the requirement for the recommended phase II dose. On Schedule 1, 1 of the 11 patients treated with 1000 mg experienced a DLT (Grade 2 intolerable maculopapular rash), and one of three patients treated with 1250 mg experienced a DLT (Grade 3 dermatitis acneiform). However, at the 1250 mg dose, the toxicity was progressive and all three patients experienced DLT-equivalent toxicities including Grade 3 pustular rash in one patient, and Grade 3 dermatitis acneiform and Grade 4 hypomagnesemia in one patient. Therefore, 1250 mg was determined to exceed the MTD, and the MTD for the Q2W treatment was determined to be 1000 mg.

On Schedule 2, no DLTs or DLT-equivalent toxicities were observed in the five patients treated at a dose of 500 mg; however, 1 DLT (Grade 3 fatigue) and 3 DLT-equivalent toxicities (Grade 3 fatigue, Grade 3 dermatitis acneiform, and Grade 2 mucosal inflammation/maculopapular rash) were observed in a total of four patients who received 600 mg. As a result, the MTD for Schedule 2 was determined to be 500 mg.

### Pharmacokinetics

Pharmacokinetic data were available from all 29 patients who received at least 1 dose of study drug on either Schedule 1 or 2. The concentration–time profiles were superimposable across both schedules of administration, and the EGFR and MET bioanalytical assays had a high degree of concordance. This evidence illustrates the in vivo stability of the bispecific antibody during the PK sampling period in Cycles 1 and 2 (Online Resources 1–4).

#### Schedule 1

**Fig. 2:**
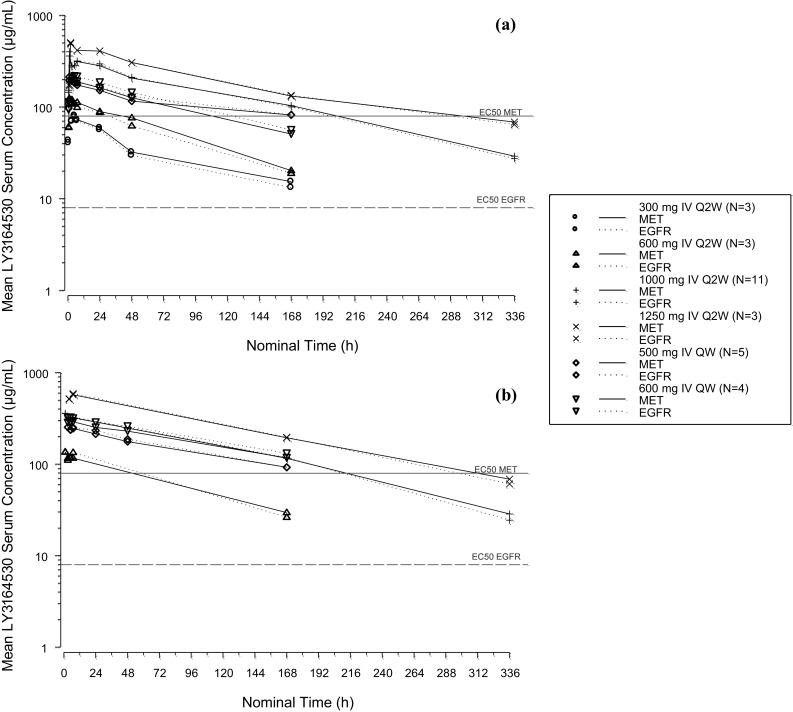
LY3164530 mean serum concentration–time plots based on the MET- and EGFR-specific ELISA assays from Day 1 (**a**) to Day 15 (Q2W) or Day 22 (QW) (**b**) of Cycle 1 for Schedules 1 and 2 (*N* = 29). *EC50* half-maximal effective serum concentration, *EGFR* epidermal growth factor receptor, *ELISA* enzyme-linked immunosorbent assay, *IV* intravenous, *MET* mesenchymal–epithelial transition factor, *N* number of patients, *Q2W* once every 2 weeks, *QW* once weekly

**Fig. 3:**
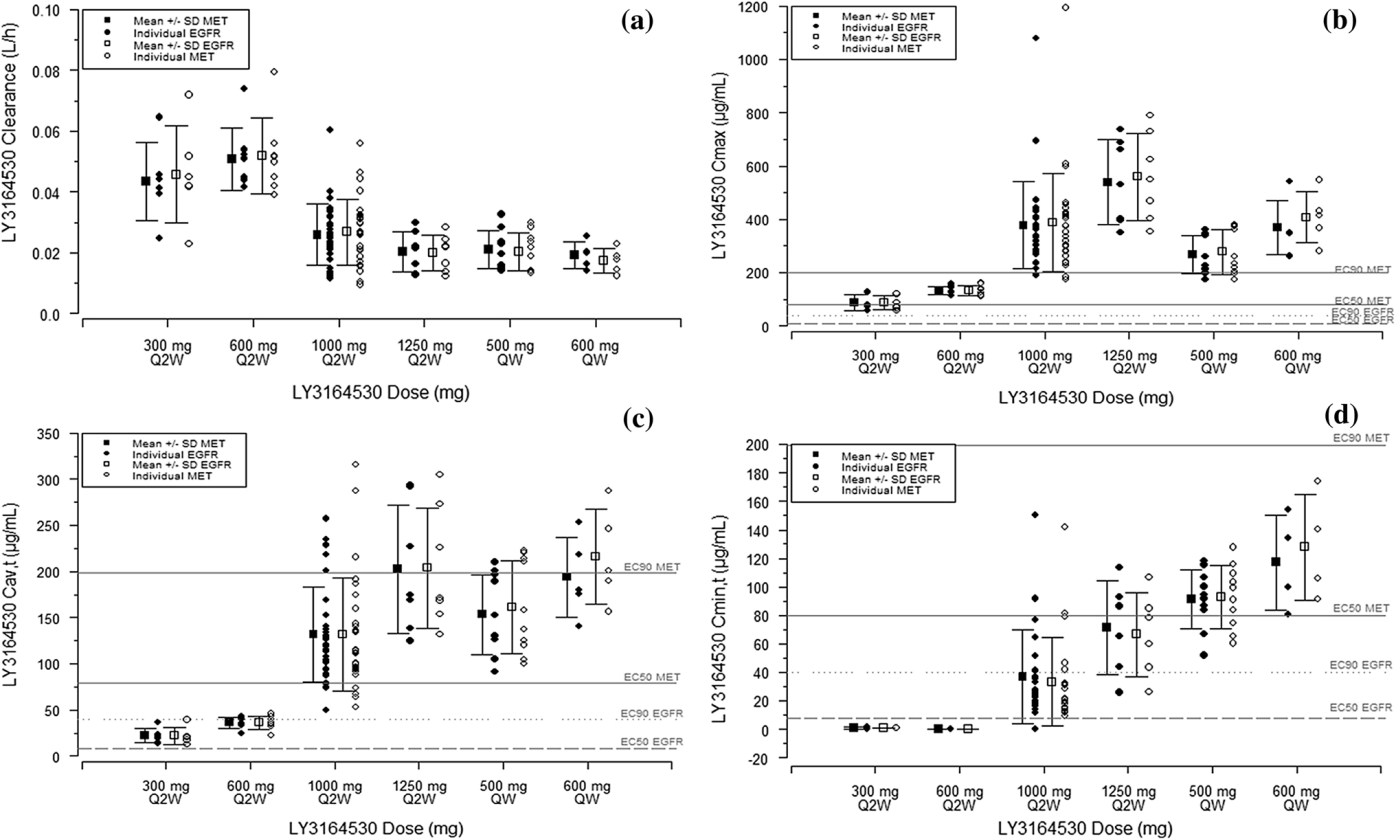
LY3164530 pharmacokinetic analysis. LY3164530 clearance (**a**), *C*_max_ (**b**), *C*_av,τ_ (**c**), and *C*_min,τ_ (**d**) individual and arithmetic mean (± SD) values combined from Day 1 to Day 15 of Cycle 1 and Day 1 of Cycle 2 across dose levels for Schedule 1 (Q2W *N* = 20) and from Day 22 of Cycle 1 and Day 1 of Cycle 2 for Schedule 2 (QW *N* = 9) that are based on the MET- and EGFR-specific assays. Horizontal lines indicate the MET and EGFR EC_50_ and EC_90_ values. *AUC*_*0–τ*_, area under the serum concentration versus time curve over the dosing interval, *C*_*av,τ*_ average serum concentration over dosing interval *(τ)* calculated using AUC_0–τ_, *C*_*max*_ maximum serum concentration, *C*_*min*_,_*τ*_ minimum serum concentration over dosing interval *(τ), EC*_*50*_ half-maximal effective serum concentration, *EC*_*90*_ 90% of maximal effective serum concentration, *EGFR* epidermal growth factor receptor, *MET* mesenchymal–epithelial transition factor, *Q2W* once every 2 weeks, *QW* once weekly, *SD* standard deviation

The *C*_max_ occurred at ~ 3 h (*t*_max_) following the start of infusion, and serum concentrations declined in a monoexponential manner (Fig. 2). Dose-dependent increases in the systemic exposure of LY3164530 were observed following single and multiple doses across the dose range (Figs. 2, 3). The CL decreased by ~ 50% when LY3164530 was escalated from 600 to 1000 mg, indicating the saturation of cell-surface receptors by study drug and a slower non-receptor-mediated clearance predominated at doses > 600 mg on Schedule 1. At doses of 1000 and 1250 mg, the mean elimination half-life (*t*_1/2_) was ~ 104 h (~ 4 days), which is ~ 39 h longer on average than the *t*_1/2_ at doses of 300 and 600 mg (mean *t*_1/2_ ~ 65 h) (Online Resources 1–4). Furthermore, a minor amount of accumulation in serum was observed within Cycle 1 (mean intracycle accumulation ratio of ~ 1.4) and between Cycles 1 and 2 (mean intercycle accumulation ratio of ~ 1.1) across all doses with Schedule 1.

The *C*_max_ following a 1000 mg dose (mean *C*_max_ range 346–367 µg/mL) and the average serum concentration over the dosing interval (*C*_av,τ_) (mean *C*_av,τ_ range 110–133 µg/mL) in Cycles 1 and 2 were greater than both the EGFR half-maximal effective serum concentration (EC_50_) (7.95 µg/mL) and MET EC_50_ (79.5 µg/mL) (Fig. 3). However, the minimum serum concentration over the dosing interval (*C*_min,τ_) following 1000 mg on Schedule 1 of LY3164530 in Cycles 1 and 2 (mean *C*_min,τ_ range 17.6–40.8 µg/mL) was lower than the MET EC_50_ (79.5 µg/mL), which is the predicted target concentration to maintain throughout the dosing interval to achieve the pharmacologic activity of LY3164530. Therefore, a weekly schedule of administration (Schedule 2) was tested to try to minimize the fluctuation between the *C*_min_ and *C*_max_ and thereby sustain a higher and more consistent level of inhibition of both EGFR and MET.

#### Schedule 2

The *C*_max_ occurred at ~ 2 h (*t*_max_) following the start of infusion of 500 and 600 mg doses, and serum concentrations declined in a monoexponential manner. A moderate amount of accumulation within Cycle 1 and between Cycles 1 and 2 (mean accumulation ratios ~ 1.7) was observed across doses on Schedule 2, and the CL following 500 mg on Schedule 2 was the same as that seen with the 1000 mg on Schedule 1 (Online Resources 1–4). Across Cycles 1 and 2, the mean *C*_min,τ_ (∼ 90 µg/mL) was greater than the EGFR EC_90_ (38.9 µg/mL) and MET EC_50_ (79.5 µg/mL), while the mean *C*_av,τ_ (∼ 151 µg/mL) on Day 22 of Cycle 1 and Day 1 of Cycle 2 was higher than that observed with Schedule 1 (110–133 µg/mg). Although a higher systemic exposure was achieved with 500 mg on Schedule 2 compared with 1000 mg on Schedule 1 (Fig. 3), neither schedule exceeded the MET EC_90_ (199 µg/ml). The mean *t*_1/2_ at both doses administered weekly (Schedule 2) increased from approximately 82 h (~ 3.4 days) on Day 1 of Cycle 1 to ~ 111 h (~ 4.6 days) following repeat administration (i.e., Day 22 of Cycle 1 and Day 1 of Cycle 2), consistent with a decrease in CL following repeat administration (Fig. 3, Online Resources 1–4). The average *t*_1/2_ of LY3164530 after repeat administration on Schedule 2 was approximately equal to the average *t*_1/2_ at doses > 600 mg on Schedule 1.

### Safety

**Fig. 4:**
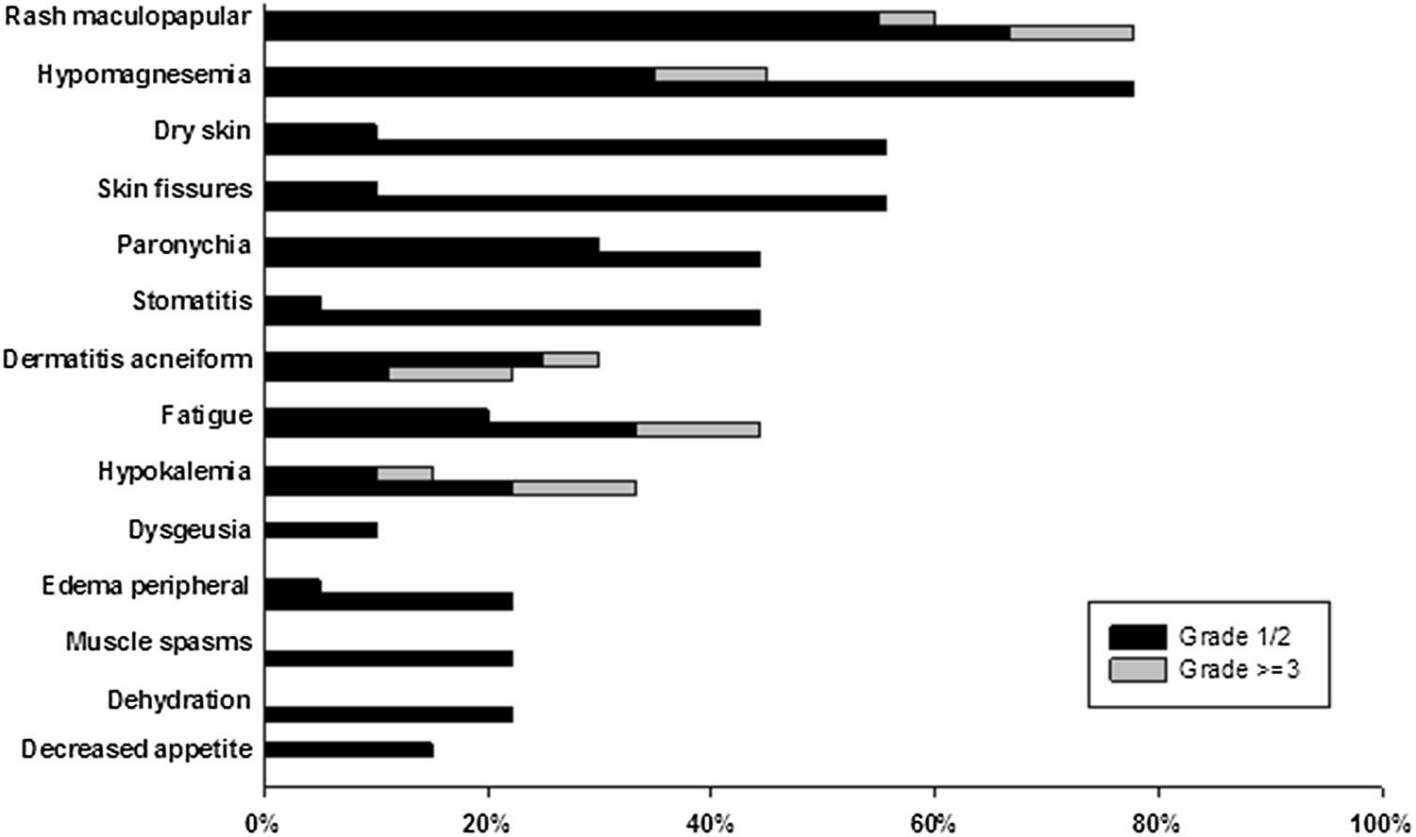
Summary of TEAEs by maximum CTCAE Grade and preferred term related to study treatment. TEAEs in ≥ 10% of the patients, in > 1 patient regardless of Schedule are shown per Schedule (top bar = Schedule 1, bottom bar = Schedule 2) and by combined Grade (1 + 2 and ≥ 3). *CTCAE* Common terminology criteria for adverse events, *TEAE* treatment-emergent adverse effect

The most frequent TEAEs (in ≥ 10% of the patients) by severity and relatedness to study drug are listed in Online Resource 5. The most frequent TEAEs (in ≥ 20% of the patients) related to study drug included maculopapular rash/dermatitis acneiform (83%, Grade 3/4 17%), hypomagnesemia (55.2%, Grade 3/4 6.9%), paronychia (34.5%), fatigue (27.6%, Grade 3/4 3.4%), dry skin (24.1%), skin fissures (24.1%), and hypokalemia (20.7%, Grade 3/4 6.9%) (Fig. 4). No patients died while on study treatment. One patient died during the follow-up period due to PD. A total of five patients experienced eight serious adverse events (SAEs); these included dysphagia (two events in a single patient), urosepsis, pneumonia, cellulitis, pneumonia aspiration, hypotension, and a Grade 1 hypomagnesemia at baseline that worsened to Grade 2. The worsening of hypomagnesemia occurred in a patient with metastatic cutaneous squamous cell carcinoma who had received 1000 mg on Schedule 1. Of the 8 SAEs, this was the only SAE considered possibly related to study drug. No infusion-related reactions were reported, and treatment-emergent ADA were not detected.

### Efficacy

The overall response rate was 10.3% and the disease control rate was 51.7; 17.2% of patients had SD ≥ 4 months (Table 1**)**.The best overall response achieved was PR in three patients, all of whom were on Schedule 2 (two patients with CRC treated with 500 mg, 5 and 6 cycles on treatment, respectively) and one patient with NOS SCC treated with 600 mg (eight cycles on treatment). Two of these patients discontinued the study due to withdrawal by subject and 1 due to PD. SD was observed in 9 (45%) patients on Schedule 1 and 3 (33.3%) patients on Schedule 2 (Table 1). For patients on Schedule 1, the median duration of SD was 1.9 months (range 0.9–7.4 months); for patients on Schedule 2, the median duration of SD was 3.5 months (range 1.4–10.6 months). Two patients on Schedule 1 with a best overall response of SD had durations of SD > 4 months (5.9 and 7.4 months), whereas the three patients on Schedule 2 with a best overall response of PR had a duration of SD > 4 months (5.8, 7.2, and 10.6 months).

**Table 1:** Summary of best overall response

	Schedule 1 (*n* = 20)	Schedule 2 (*n* = 9)	Total (*N* = 29)
Best overall response, *n* (%)			
Complete response (CR)	0	0	0
Partial response (PR)	0	3 (33.3)	3 (10.3)
Stable disease (SD) > 2 cycles	9 (45.0)	3 (33.3)	12 (41.4)
SD ≥ 4 cycles	2 (10.0)	3 (33.3)	5 (17.2)
Progressive disease (PD)	9 (45.0)	3 (33.3)	12 (41.4)
Missing	2 (10.0)	0	2 (6.9)
Overall response rate (CR/PR), *n* (%)	0	3 (33.3)	3 (10.3)
Disease control rate (CR/PR/SD), *n* (%)	9 (45.0)	6 (66.7)	15 (51.7)

Response criteria used was RECIST v1.1

*N* Number of patients in population, *n* number of patients, *RECIST* response evaluation criteria in solid tumor

### Predictive markers

**Fig. 5:**
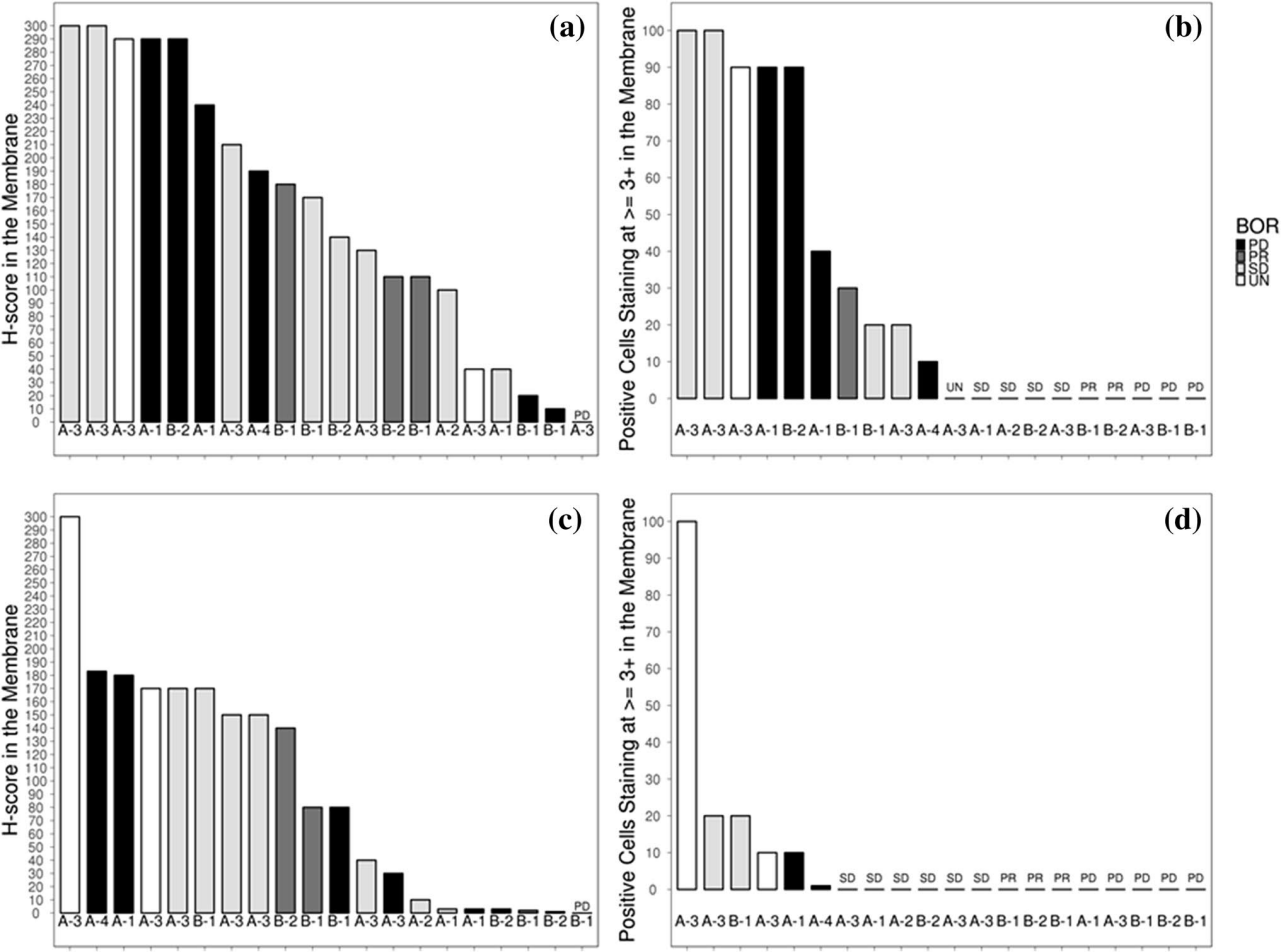
Baseline MET and EGFR expression via immunohistochemistry of tumor samples. Baseline MET expression (H-score) by BOR (**a**), baseline MET expression (% intensity 3+) by BOR (**b**), baseline EGFR expression (H-score) by BOR (**c**), and baseline EGFR expression (% intensity 3+) by BOR (**d**). Cohort listed under each bar; Cohort A = Schedule 1, Cohort B = Schedule 2. Cohort A-1 received a 300 mg dose; A-2 received a 600 mg dose; A-3 received a 1000 mg dose; A-4 received a 1250 mg dose; B-1 received a 500 mg dose; and B-2 received a 600 mg dose. *BOR* Best overall response, *EGFR* epidermal growth factor receptor, *MET* mesenchymal–epithelial transition factor, *PD* progressive disease, *PR* partial response, *SD* stable disease, *UN* unknown

In this small study, there were no markers identified that could predict response or resistance to LY3164530. Patients with a PR did not have the highest levels of MET expression as measured by IHC H-Score; instead, two patients with SD, two patients with PD, and one patient with an unknown response had the highest expression of MET. The median duration on therapy for the five patients with the highest expression was two cycles, and four of the patients received a dose that was at or above the recommended phase II dose for the respective schedule. None of the patients with MET intensity of 3+ in 90% of cells achieved an objective response (Fig. 5a, b). Neither EGFR expression H-score nor % intensity 3+ was predictive of response (Fig. 5c, d).

As determined by FISH, only one patient with PD who was treated with 600 mg LY3164530 weekly had a *MET* amplification. Furthermore, no patients had *KRAS*/*EGFR* somatic mutations.

## Discussion

LY3164530 was designed as a novel approach to target the interplay and corresponding resistance between the MET and EGFR pathways utilizing a single bispecific antibody. The MTD of LY3164530 for Schedules 1 and 2 were 1000 and 500 mg, respectively. Dose escalation and MTD determination was driven by an mTPI method [18]. A 3 + 3 design is commonly used in phase I trials due to its simple, intuitive, and pre-specified escalation rules. However, the 3 + 3 method has been criticized for being conservative because the method is dictated by the observed DLT rate without acknowledging the variability arising from a small cohort size [19]. Like the 3 + 3 design, the mTPI method incorporates pre-specified escalation rules. In contrast, the mTPI method is based on quantitative models that incorporate uncertainty into the decision rules, and the number of patients in each cohort is not fixed. This study effectively implemented the mTPI, and the ability to have variable cohort levels not only helped determine the MTDs, but also assess secondary and exploratory endpoints.

Prior to entering clinical testing, LY3164530 was engineered to maximize the stability of the linker to the EGFR region. The PK profiles and parameters for MET and EGFR (based on separate enzyme-linked immunosorbent assay formats) across all doses and schedules of administration demonstrated a monoexponential decline in serum concentration and a high degree of concordance, thereby confirming in vivo stability of the bispecific antibody. A slower, non-receptor-mediated clearance was observed at the MTD for each schedule of administration, indicating the saturation of cell surface receptors for EGFR and MET. However, a large fluctuation between the peak (*C*_max_) and trough serum concentrations (*C*_min,τ_) was observed with the biweekly administration (Schedule 1); therefore, weekly administration (Schedule 2) was tested to determine if a greater and more consistent inhibition of MET and EGFR could be obtained. A Schedule 2 dose of 500 mg achieved higher serum *C*_min,τ_ throughout the dosing interval compared with Schedule 1 administration of 1000 mg. In Schedule 1, the minimum serum concentration over the dosing interval was lower than the MET EC_50_, which is predicted to be the target concentration to maintain throughout the dosing interval to achieve the pharmacologic activity of LY3164530. As a result, Schedule 2 was explored and although the overall dose intensity/per cycle was the same (1000 mg biweekly versus 500 mg weekly), the weekly administration of 500 mg achieved higher serum trough concentrations (*C*_min,τ_) throughout the dosing interval compared with the biweekly schedule of administration of 1000 mg. However, although the minimum serum concentration on Schedule 2 exceeded the MET EC_50_, it did not exceed the MET EC_90_.

The most common TEAEs observed with LY3164530 included cutaneous toxicities and hypomagnesemia. These toxicities are consistent with EGFR inhibition and appeared to occur at a greater frequency and severity than that reported for cetuximab. This suggests that LY3164530 effectively inhibits EGFR since skin rash is a known pharmacodynamic marker of EGFR inhibition. This is consistent with the PK predictions that, on both Schedule 1 and 2, LY3164530 concentrations were exceeding the predicted EGFR EC_90_. Conversely, toxicities that are more often associated with MET inhibition, such as gastrointestinal toxicities observed with emibetuzumab treatment [14], were not commonly reported.

Objective clinical responses in patients with CRC (*n* = 2) or NOS SCC (*n* = 1) were observed, with three patients on Schedule 2 experiencing a PR. Each of these patients had previously received a cetuximab-containing regimen and none had achieved an objective response, although the CRC patients had a mean SD of 10 months (irinotecan/cetuximab) and 8 months (FOLFIRI/cetuximab), respectively. The patient with NOS SCC had PD following treatment with irinotecan/5FU/cetuximab. It is not known whether the responses observed in this study were due to the dual inhibition of MET/EGFR or to EGFR inhibition. To potentially inform this assessment, a key exploratory objective of the study was to investigate whether MET and/or EGFR protein expression levels or amplification, or *KRAS*/*EGFR* somatic mutations, were prognostic of LY3164530 response. Preclinical data suggested optimal activity of the bispecific antibody was likely to occur in patients with high MET expression/amplification, EGFR expression, and no *KRAS* alterations. Approximately 70% of the patients were evaluated for biomarkers at baseline, and a subset of patients were identified with high MET/EGFR expression or *MET*/*EGFR* amplification. No mutations were identified in *KRAS* or *EGFR*. Unfortunately, no discernible trends were found in baseline MET/EGFR expression, *MET*/*EGFR* amplification, or *KRAS*/*EGFR* mutation status when patients who benefited were compared with those who did not; as such, there were no biomarkers identified that predicted response or resistance to LY3164530.

The combined safety, PK, efficacy, and biomarker data lead to uncertainty around whether the extent and duration of MET inhibition was sufficient for maximal efficacy. Additionally, although the MTD was defined for each schedule, the toxicities, and in particular, the concurrent mucosal and skin effects negatively impacted the patients. Importantly, the toxicities were cumulative and those patients who withdrew consent did so after multiple cycles (range 2–10 cycles). Almost half of the patients required dose adjustments, with AEs being the most common reason for the modifications. Notably, two patients with an ongoing PR discontinued due to patient decision, secondary to ongoing AEs that did not meet the protocol-defined levels for discontinuation but impacted the patients’ quality of life. Overall, > 25% of the patients discontinued the study due to reasons other than PD (AE, subject decision, physician decision).

The dual inhibition of MET and EGFR has been explored using a combination of separate MET and EGFR inhibitors. A literature searched revealed that this is the first report of clinical data with a bispecific antibody targeting both MET and EGFR in a single molecule. Non-clinical data have been reported for other MET/EGFR bispecific antibodies including JNJ-61186372 [20], ME22S [21], and MetHer1 [22]. A limitation of the bispecific antibody approach is that stoichiometry is fixed; and, therefore, the relative inhibition of EGFR versus MET is unable to be adjusted to maximize the potential for efficacy.

A specific limitation of LY3164530 was that the target with the greatest inhibition (EGFR) was also the target with a lower threshold for toxicity. It may have been preferable to have greater MET inhibition with LY3164530, since MET inhibitors have reported a more tolerable toxicity profile than EGFR inhibitors. During dose escalation, this may have allowed both targets to be inhibited above their predicted EC_90_. In addition, post-treatment biopsies to assess pharmacodynamic effects were not collected and circulating markers (e.g. HGF and TGFα) were not informative (data not shown).

Although the MTD of LY3164530 on each schedule was identified, given the toxicity, dose adjustments, PK data, limited efficacy, and inability to prospectively select patients most likely to respond to LY3164530, the molecule will not advance to phase II development. Nonetheless, the present results demonstrate the ability of the bispecific antibody to effectively engage targets and produce objective clinical responses. Optimizing the balance between the inhibition of pertinent targets relative to their toxicity profile may help improve upon the efficacy and safety of future bispecific antibodies targeting these agents. The next generation of bispecific antibodies may provide further insight into the utility of targeting the resistance and crosstalk that occurs between MET and EGFR.

## Electronic supplementary material

Below is the link to the electronic supplementary material.

Supplementary material 1 (DOCX 55 KB)

## References

[1] Hartmann S, Bhola NE, Grandis JR (2016). HGF/Met signaling in head and neck cancer: impact on the tumor microenvironment. Clin Cancer Res.

[2] Nanjo S, Yamada T, Nishihara H, Takeuchi S, Sano T, Nakagawa T, Ishikawa D, Zhao L, Ebi H, Yasumoto K, Matsumoto K, Yano S (2013). Ability of the Met kinase inhibitor crizotinib and new generation EGFR inhibitors to overcome resistance to EGFR inhibitors. PLoS One.

[3] Qamsari ES, Sharifzadeh Z, Bagheri S, Riazi-Rad F, Younesi V, Abolhassani M, Ghaderi SS, Baradaran B, Somi MH, Yousefi M (2017). Isolation and characterization of anti c-met single chain fragment variable (scFv) antibodies. J Immunotoxicol.

[4] Seshacharyulu P, Ponnusamy MP, Haridas D, Jain M, Ganti AK, Batra SK (2012). Targeting the EGFR signaling pathway in cancer therapy. Expert Opin Ther Targets.

[5] Gherardi E, Birchmeier W, Birchmeier C, Vande Woude G (2012). Targeting MET in cancer: rationale and progress. Nat Rev Cancer.

[6] Graveel CR, Tolbert D, Vande Woude GF (2013). MET: a critical player in tumorigenesis and therapeutic target. Cold Spring Harb Perspect Biol.

[7] Remon J, Morán T, Majem M, Reguart N, Dalmau E, Márquez-Medina D, Lianes P (2014). Acquired resistance to epidermal growth factor receptor tyrosine kinase inhibitors in EGFR-mutant non-small cell lung cancer: a new era begins. Cancer Treat Rev.

[8] Chong CR, Janne PA (2013). The quest to overcome resistance to EGFR-targeted therapies in cancer. Nat Med.

[9] McDermott U, Pusapati RV, Christensen JG, Gray NS, Settleman J (2010). Acquired resistance of non-small cell lung cancer cells to MET kinase inhibition is mediated by a switch to epidermal growth factor receptor dependency. Cancer Res.

[10] Engelman JA, Zejnullahu K, Mitsudomi T, Song Y, Hyland C, Park JO, Lindeman N, Gale CM, Zhao X, Christensen J, Kosaka T, Holmes AJ, Rogers AM, Cappuzzo F, Mok T, Lee C, Johnson BE, Cantley LC, Jänne PA (2007). MET amplification leads to gefitinib resistance in lung cancer by activating ERBB3 signaling. Science.

[11] Yano S, Yamada T, Takeuchi S, Tachibana K, Minami Y, Yatabe Y, Mitsudomi T, Tanaka H, Kimura T, Kudoh S, Nokihara H, Ohe Y, Yokota J, Uramoto H, Yasumoto K, Kiura K, Higashiyama M, Oda M, Saito H, Yoshida J, Kondoh K, Noguchi M (2011). Hepatocyte growth factor expression in EGFR mutant lung cancer with intrinsic and acquired resistance to tyrosine kinase inhibitors in a Japanese cohort. J Thorac Oncol.

[12] Garajová I, Giovannetti E, Biasco G, Peters GJ (2015). c-Met as a target for personalized therapy. Transl Oncogenom.

[13] Eng C, Bessudo A, Hart LL, Severtsev A, Gladkov O, Muller L, Kopp ML, Vladimirov V, Langdon R, Kotiv B, Barni S, Hsu C, Bolotin E, von Roemeling R, Schwartz B, Bendell JC (2016). A randomized, placebo-controlled, phase 1/2 study of tivantinib (ARQ 197) in combination with irinotecan and cetuximab in patients with metastatic colorectal cancer with wild-type KRAS who have received first-line systemic therapy. Int J Cancer.

[14] Rosen LS, Goldman JW, Algazi AP, Turner PK, Moser B, Hu T, Wang XA, Tuttle J, Wacheck V, Wooldridge JE, Banck M (2017). A first-in-human phase I study of a bivalent MET antibody, emibetuzumab (LY2875358), as monotherapy and in combination with erlotinib in advanced cancer. Clin Cancer Res.

[15] Rhoden JJ, Dyas GL, Wroblewski VJ (2016). A modeling and experimental investigation of the effects of antigen density, binding affinity, and antigen expression ratio on bispecific antibody binding to cell surface targets. J Biol Chem.

[16] Eli Lilly and Company (2016) Erbitux® (cetuximab). Highlights of prescribing information. http://pi.lilly.com/us/erbitux-uspi.pdf. Accessed 16 Mar 2018

[17] Liu L, Zeng W, Chedid M, Zeng Y, Tschang S, Tian Tang Y, Lu JY (2016). A novel MET-EGFR bispecific antibody LY3164530 shows advantage over combining MET and EGFR antibodies in tumor inhibition and overcome resistance. AACR; Cancer Res.

[18] Ji Y, Wang SJ (2013). Modified toxicity probability interval design: a safer and more reliable method than the 3+3 design for practical phase I trials. J Clin Oncol.

[19] Ursino M, Zohar S, Lentz F, Alberti C, Friede T, Stallard N, Comets E (2017). Dose-finding methods for Phase I clinical trials using pharmacokinetics in small populations. Biom J.

[20] Moores SL, Chiu ML, Bushey BS, Chevalier K, Luistro L, Dorn K, Brezski RJ, Haytko P, Kelly T, Wu SJ, Martin PL, Neijssen J, Parren PW, Schuurman J, Attar RM, Laquerre S, Lorenzi MV, Anderson GM (2016). A novel bispecific antibody targeting EGFR and cMet is effective against EGFR inhibitor-resistant lung tumors. Cancer Res.

[21] Lee JM, Lee SH, Hwang JW, Oh SJ, Kim B, Jung S, Shim SH, Lin PW, Lee SB, Cho MY, Koh YJ, Kim SY, Ahn S, Lee J, Kim KM, Cheong KH, Choi J, Kim KA (2016). Novel strategy for a bispecific antibody: induction of dual target internalization and degradation. Oncogene.

[22] Castoldi R, Ecker V, Wiehle L, Majety M, Busl-Schuller R, Asmussen M, Nopora A, Jucknischke U, Osl F, Kobold S, Scheuer W, Venturi M, Klein C, Niederfellner G, Sustmann C (2013). A novel bispecific EGFR/Met antibody blocks tumor-promoting phenotypic effects induced by resistance to EGFR inhibition and has potent antitumor activity. Oncogene.

