# Supplementary material for: A phase I study of LY3164530, a bispecific antibody targeting MET and EGFR, in patients with advanced or metastatic cancer
Source: Cancer Chemother Pharmacol. 2018 Jun 20;82(3):407–18. doi: 10.1007/s00280-018-3623-7 (PMC6105165; doi:10.1007/s00280-018-3623-7)
Supplement: Supplementary file 1 — Supplementary material 1 (DOCX 55 KB) [file 280_2018_3623_MOESM1_ESM.docx]

**Appendix**

Online Resource 1 LY3164530 non-compartmental PK parameter estimates from Cycles 1 and 2 based on the EGFR-specific ELISA assay for all patients with available PK data from Schedule 1 (Q2W) (N=20)

|  | **EGFR-Specific ELISA Assay** | | | | | | | | | | | |
| --- | --- | --- | --- | --- | --- | --- | --- | --- | --- | --- | --- | --- |
|  | **Geometric Mean (CV%)** | | | | | | | | | | | |
| **Dose** | **300 mg Q2W** | | | **600 mg Q2W** | | | **1000 mg Q2W** | | | **1250 mg Q2W** | | |
| **Cycle** | **1** | | **2** | **1** | | **2** | **1** | | **2** | **1** | | **2** |
| **Day** | **1** | **15** | **1** | **1** | **15** | **1** | **1** | **15** | **1** | **1** | **15** | **1** |
| **N** | 3 | 2^a^ | 1^a^ | 3 | 3 | 2^a^ | 11 | 11 | 9 | 3 | 3 | 1^a^ |
| **C_max_**  **(μg/mL)** | 85.1  (38) | 119.55,  66.36 | 72.19 | 122  (12) | 147  (15) | 115.02,  135.38 | 367  (52) | 348  (34) | 357  (35) | 496  (30) | 615  (38) | 468.9 |
| **t_max_^b^**  **(hr)** | 3  (1.12–5) | 3,  7.25 | 1.05 | 3  (1.02–3.08) | 5.03  (1–7.17) | 5.03,  5.05 | 5.08  (1–24.58) | 1.32  (1–7.10) | 3  (1–5.08) | 2.02  (2–4.57) | 4.08  (2.08–8.12) | 2.05 |
| **C_av,τ_**  **(μg/mL)** | 16.6  (28) | 39.0,  21.3 | 19.9 | 29.1  (23) | 40.8  (15) | 35.9,  39.7 | 110  (42) | 127  (39)^c^ | 128  (55)^d^ | 171  (28) | 242  (31) | 153 |
| **C_min,τ_**  **(μg/mL)** | 0.898^e^ | NA | NA | NA | NA | NA | 22.0  (73)^c^ | 25.5  (38)^f^ | 35.4  (142)^g^ | 55.2  (80) | 66.0  (38) | NA |
| **AUC_0-τ_**  **(μg⋅hr/mL)** | 5580  (28) | 13100,  7170 | 6690 | 9780  (23) | 13700  (15) | 12100,  13300 | 37000  (42) | 42700  (39)^c^ | 43000  (55)^d^ | 57500  (28) | 81400  (31) | 51500 |
| **CL**  **(L/hr)** | 0.054  (28) | 0.023,  0.042 | 0.045 | 0.061  (23) | 0.044  (15) | 0.050,  0.045 | 0.027  (42) | 0.023  (39)^c^ | 0.023  (55)^d^ | 0.022  (28) | 0.015  (31) | 0.024 |
| **V_ss_**  **(L)** | 4.74  (31) | 3.01,  4.58 | 4.40 | 5.73  (12) | 4.54  (16) | 5.25,  4.45 | 3.39  (32) | 3.37  (35^c^) | 3.23  (30)^d^ | 3.56  (4) | 2.55  (29) | 3.51 |
| **t_1/2_**  **(hr)** | 58.6  (18) | 89.7,  76.4 | 69.1 | 65.0  (15) | 71.8  (12) | 73.4,  68.0 | 85.1  (27) | 100  (27)^c^ | 96.5  (27)^d^ | 113  (26) | 116  (8) | 101 |

*AUC_0-τ_* area under the serum concentration–time curve over the dosing interval from time 0 to 336 hours (τ), *C_av,τ_* average serum concentration over dosing interval (τ) calculated using AUC_0-τ_, *CL* total systemic clearance, *C_max_* maximum serum concentration, *C_min,τ_* minimum serum concentration over dosing interval (τ); *CV%* percent coefficient of variation; *EGFR* epidermal growth factor receptor; *ELISA* enzyme-linked immunosorbent assay, *N* number of patients, *NA* Not applicable, *PK* pharmacokinetics, *Q2W* once every 2 weeks, *t_1/2_* elimination half-life, *t_max_* time of maximum serum concentration, V_ss_ volume of distribution at steady state

^a^ Reported as individual patient parameter values

^b^ Median (Minimum – Maximum)

^c^ N=10; ^d^ N=8; ^e^ N=1; ^f^ N=7; ^g^ N=5

Online Resource 2 LY3164530 non-compartmental PK parameter estimates in Cycles 1 and 2 based on the MET-specific ELISA assay for all patients with available PK data from Schedule 1 (Q2W) (N=20)

|  | **MET-Specific ELISA Assay** | | | | | | | | | | | |
| --- | --- | --- | --- | --- | --- | --- | --- | --- | --- | --- | --- | --- |
|  | **Geometric Mean (CV%)** | | | | | | | | | | | |
| **Dose** | **300 mg Q2W** | | | **600 mg Q2W** | | | **1000 mg Q2W** | | | **1250 mg Q2W** | | |
| **Cycle** | **1** | | **2** | **1** | | **2** | **1** | | **2** | **1** | | **2** |
| **Day** | **1** | **15** | **1** | **1** | **15** | **1** | **1** | **15** | **1** | **1** | **15** | **1** |
| **N** | 3 | 2^a^ | 1^a^ | 3 | 3 | 2^a^ | 11 | 11 | 9 | 3 | 3 | 1^a^ |
| **C_max_**  **(μg/mL)** | 82.5  (41) | 127.21,  73.21 | 72.32 | 124  (15) | 136  (12) | 123.84, 143.68 | 365  (46) | 346  (33) | 353  (27) | 498  (33) | 587  (35) | 400.9 |
| **t_max_^b^**  **(hr)** | 1.30  (1.12–5.0) | 3.0,  7.25 | 1.05 | 1.03  (1–6.97) | 1.08  (1–5) | 1.07, 5.05 | 5.12  (1–24.58) | 1.32  (1.02–7.10) | 4.97  (1–5.08) | 4.57  (2–6.07) | 4.08  (2.08–8.12) | 2.05 |
| **C_av,τ_**  **(μg/mL)** | 18.3  (26) | 36.2,  21.6 | 20.2 | 32.3  (27) | 38.5  (14) | 35.1, 39.7 | 112  (41) | 128  (34) | 133  (45)^c^ | 171  (31) | 244  (33) | 138 |
| **C_min,τ_**  **(μg/mL)** | 1.519^d^ | 0.158^d^ | NA | NA | 0.343^d^ | 0.257, 0.145 | 17.6  (309) | 29.5  (46)^e^ | 40.8  (133)^f^ | 57.8  (86) | 70.7  (43) | NA |
| **AUC_0-τ_**  **(μg⋅hr/mL)** | 6150  (26) | 12200,  7260 | 6800 | 10900  (27) | 12900  (14) | 11800, 13300 | 37500  (41) | 43000  (34) | 44700  (45)^c^ | 57300  (31) | 82000  (33) | 46400 |
| **CL**  **(L/hr)** | 0.049  (26) | 0.025,  0.041 | 0.044 | 0.055  (27) | 0.046  (14) | 0.051, 0.045 | 0.027  (41) | 0.023  (34) | 0.022  (45)^c^ | 0.022  (31) | 0.016  (33) | 0.027 |
| **V_ss_**  **(L)** | 4.61  (25) | 2.84,  3.16 | 4.55 | 5.27  (20) | 4.84  (22) | 4.11, 3.26 | 3.58  (29) | 3.43  (24) | 3.39  (25)^c^ | 3.65  (1) | 2.67  (28) | 3.72 |
| **t_1/2_**  **(hr)** | 63.1  (12) | 77.9,  36.8 | 71.9 | 66.3  (14) | 66.7  (44) | 44.2, 33.1 | 89.4  (39) | 103  (25) | 105  (35)^c^ | 116  (29) | 123  (6) | 96.5 |

*AUC_0-τ_* area under the serum concentration–time curve over the dosing interval from time 0 to 336 hours (τ), *C_av,τ_* average serum concentration over dosing interval (τ) calculated using AUC_0-τ_, *CL* total systemic clearance, *C_max_* maximum serum concentration, *C_min,τ_* minimum serum concentration over dosing interval (τ), *CV%* percent coefficient of variation, *ELISA* enzyme-linked immunosorbent assay, *h* hours, *MET* mesenchymal–epithelial transition factor, *N* number of patients, *NA* Not applicable, *PK* pharmacokinetics, *t_1/2_* elimination half-life, *t_max_* time of maximum serum concentration, *V_ss_* volume of distribution at steady state.

^a^ Reported as individual patient parameter values

^b^ Median (Minimum – Maximum)

^c^ N=8; ^d^ N=1; ^e^ N=7; ^f^ N=5

Online Resource 3 LY3164530 non-compartmental PK parameter estimates in Cycles 1 and 2 based on the EGFR-specific ELISA assay for all patients with available PK data from Schedule 2 (QW) (N=9)

|  | **EGFR-Specific ELISA Assay** | | | | | |
| --- | --- | --- | --- | --- | --- | --- |
|  | **Geometric Mean (CV%)** | | | | | |
| **Dose** | **500 mg QW** | | | **600 mg QW** | | |
| **Cycle** | **1** | | **2** | **1** | | **2** |
| **Day** | **1** | **22** | **1** | **1** | **22** | **1** |
| **N** | 5 | 5 | 5 | 4 | 3 | 2^a^ |
| **C_max_**  **(**μ**g/mL)** | 226  (20) | 262  (35) | 270  (32) | 255  (21) | 350  (20) | 547.9,  431.2 |
| **t_max_^b^**  **(hr)** | 1.13  (1.07–7) | 3.02  (1.02–7.05) | 5.00  (1.02–7.05) | 3.04  (0.35–7.07) | 1.07  (1.03–1.28) | 3.02,  1.03 |
| **C_av,τ_**  **(**μ**g/mL)** | 99.5  (37)^c^ | 152  (35) | 156  (35) | 118  (21) | 208  (31) | 190,  246 |
| **C_min,τ_**  **(μg/mL)** | 30.9  (903)^c^ | 89.4  (28) | 91.7  (25) | 52.4  (46) | 105.7,^a,d^  174.0^a,d^ | 91.2,  140.5 |
| **AUC_0-τ_**  **(μg⋅hr/mL)** | 16700  (37)^c^ | 25500  (35) | 26300  (35) | 19800  (21) | 34900  (31) | 31900,  41300 |
| **CL**  **(L/hr)** | 0.030  (37)^c^ | 0.020  (35) | 0.019  (35) | 0.030  (21) | 0.017  (31) | 0.019,  0.015 |
| **V_ss_**  **(L)** | 3.07  (40)^c^ | 3.29  (51) | 3.24  (57) | 3.77  (17) | 2.83  (30) | 2.37,  2.27 |
| **t_1/2_**  **(hr)** | 68.4  (91)^c^ | 116  (17) | 118  (25) | 86.4  (28) | 114  (17) | 87.4,  108 |

*AUC_0-τ_* area under the serum concentration–time curve over the dosing interval from time 0 to 168 hours (τ), *C_av,τ_* average serum concentration over dosing interval (τ) calculated using AUC_0-τ_, *CL* total systemic clearance, *C_max_* maximum serum concentration, *C_min,τ_* minimum serum concentration over dosing interval (τ), *CV%* percent coefficient of variation, *EGFR* epidermal growth factor receptor, *ELISA* enzyme-linked immunosorbent assay, *N* number of patients, *PK* pharmacokinetics, *QW* once weekly, *t_1/2_* elimination half-life, *t_max_* time of maximum serum concentration, *V_ss_* volume of distribution at steady state

^a^ Reported as individual patient parameter values

^b^ Median (Minimum – Maximum)

^c^ N=4

^d^ N=2

Online Resource 4 LY3164530 non-compartmental PK parameter estimates in Cycles 1 and 2 based on the MET-specific ELISA Assay for all patients with available PK data from Schedule 2 (QW) (N=9)

|  | **MET-Specific ELISA Assay** | | | | | |
| --- | --- | --- | --- | --- | --- | --- |
|  | **Geometric Mean (CV%)** | | | | | |
| **Dose** | **500 mg QW** | | | **600 mg QW** | | |
| **Cycle** | **1** | | **2** | **1** | | **2** |
| **Day** | **1** | **22** | **1** | **1** | **22** | **1** |
| **N** | 5 | 5 | 5 | 4 | 3 | 2^a^ |
| **C_max_**  **(μg/mL)** | 208  (23) | 260  (32) | 258  (27) | 221  (21) | 317  (17) | 541.5,  347.0 |
| **t_max_^b^**  **(hr)** | 3.02  (1.13–7) | 3.02  (1.02–7.05) | 1.08  (1.02–7.05) | 1.15  (0.35–7.07) | 1.28  (1.03–7.02) | 3.02,  1.03 |
| **C_av,τ_**  **(μg/mL)** | 97.1  (22) | 148  (29) | 148  (34) | 104  (20) | 186  (30) | 176,  218 |
| **C_min,τ_**  **(μg/mL)** | 63.4  (90)^c^ | 90.5  (23) | 87.7  (31) | 47.4  (46) | 99.5  154^a^ | 80.7,  134.1 |
| **AUC_0-τ_**  **(μg⋅hr/mL)** | 16300  (22) | 24800  (29) | 24800  (34) | 17500  (20) | 31200  (30) | 29500,  36700 |
| **CL**  **(L/hr)** | 0.031  (22) | 0.020  (29) | 0.020  (34) | 0.034  (20) | 0.019  (30) | 0.020,  0.016 |
| **V_ss_**  **(L)** | 3.71  (24) | 3.54  (44) | 3.52  (51) | 4.41  (23) | 3.27  (31) | 2.42,  2.98 |
| **t_1/2_**  **(hr)** | 83.2  (12) | 121  (17) | 120  (28) | 89.5  (33) | 118  (16) | 82.7,  126 |

*AUC_0-τ_* area under the serum concentration–time curve over the dosing interval from time 0 to 168 hours (τ), *C_av,τ_* average serum concentration over dosing interval (τ) calculated using AUC_0-τ_, *CL* total systemic clearance, *C_max_* maximum serum concentration, *C_min,τ_* minimum serum concentration over dosing interval (τ), *CV%* percent coefficient of variation, *ELISA* enzyme-linked immunosorbent assay, *MET* mesenchymal–epithelial transition factor, *N* number of patients, *PK* pharmacokinetics, *QW* once weekly, *t_1/2_* elimination half-life, *t_max_* time of maximum serum concentration, *V_ss_* volume of distribution at steady state

^a^ Reported as individual patient parameter values

^b^ Median (Minimum – Maximum)

^c^ N=4

**Online Resource 5 Summary of treatment-emergent adverse events ≥20% in both schedules, by severity and relatedness to study treatment (safety population)**

| MedDRA Preferred Term | Schedule 1 (n=20) | | | | Schedule 2 (n=9) | | | | Total (N=29) | |
| --- | --- | --- | --- | --- | --- | --- | --- | --- | --- | --- |
|  | All Grades | | ≥ Grade 3 | | All Grades | | ≥ Grade 3 | |  | |
|  | Not related | Possibly related | Not related | Possibly related | Not related | Possibly related | Not related | Possibly related | Not related | Possibly related |
| Patients with ≥1 TEAE, n (%) | 20  (100.0) | 18  (90.0) | 15  (75.0) | 6  (30.0) | 9  (100.0) | 9  (100.0) | 5  (55.6) | 4  (44.4) | 29  (100.0) | 27  (93.1) |
| Rash maculopapular | 12  (60.0) | 12  (60.0) | 1  (5.0) | 1  (5.0) | 7  (77.8) | 7  (77.8) | 2  (22.2) | 1  (11.1) | 19  (65.5) | 19  (65.5) |
| Hypomagnesemia | 10  (50.0) | 9  (45.0) | 2  (10.0) | 2  (10.0) | 7  (77.8) | 7  (77.8) | 0 | 0 | 17  (58.6) | 16  (55.2) |
| Fatigue | 6  (30.0) | 4  (20.0) | 0 | 0 | 5  (55.6) | 4  (44.4) | 2  (22.2) | 1 (11.1) | 11  (37.9) | 8  (27.7) |
| Edema peripheral | 5  (25.0) | 1  (5.0) | 0 | 0 | 5  (55.6) | 2  (22.2) | 0 | 0 | 10  (34.5) | 3  (10.3) |
| Paronychia | 6  (30.0) | 6  (30.0) | 0 | 0 | 4  (44.4) | 4  (44.4) | 0 | 0 | 10  (34.5) | 10 (34.5) |
| Dry skin | 2  (10.0) | 2  (10.0) | 0 | 0 | 7  (77.8) | 5  (55.6) | 0 | 0 | 9  (31.0) | 7  (24.1) |
| Dyspnea | 6  (30.0) | 1 (5.0) | 1  (5.0) | 0 | 3  (33.3) | 0 | 1  (11.1) | 0 | 9  (31.0) | 1 (3.4) |
| Dermatitis acneiform | 6  (30.0) | 6  (30.0) | 1  (5.0) | 1  (5.0) | 2  (22.2) | 2  (22.2) | 1  (11.1) | 1  (11.1) | 8  (27.6) | 8  (27.7) |
| Hypokalemia | 4  (20.0) | 3  (15.0) | 1  (5.0) | 1  (5.0) | 4  (44.4) | 3  (33.3) | 1  (11.1) | 1  (11.1) | 8  (27.6) | 6  (20.7) |
| Decreased appetite | 6  (30.0) | 3  (15.0) | 0 | 0 | 1  (11.1) | 0 | 0 | 0 | 7  (24.1) | 3  (10.3) |
| Dehydration | 2  (10.0) | 0 | 1  (5.0) | 0 | 3  (33.3) | 2  (22.2) | 0 | 0 | 7  (24.1) | 2  (6.9) |
| Nausea | 4  (20.0) | 1 (5.0) | 0 | 0 | 3  (33.3) | 0 | 0 | 0 | 7  (24.1) | 1  (3.4) |
| Skin fissures | 2  (10.0) | 2  (10.0) | 0 | 0 | 5  (55.6) | 5  (55.6) | 0 | 0 | 7  (24.1) | 7  (24.1) |
| Stomatitis | 1  (5.0) | 1  (5.0) | 0 | 0 | 6  (66.7) | 4  (44.4) | 0 | 0 | 7  (24.1) | 5  (17.2) |
| Back pain | 1  (5.0) | 0 | 0 | 0 | 4  (44.4) | 1  (11.1) | 0 | 0 | 5  (17.2) | 1  (3.4) |
| Diarrhea | 3  (15.0) | 0 | 0 | 0 | 2  (22.2) | 0 | 0 | 0 | 5  (17.2) | 0 |
| Pyrexia | 2  (10.0) | 0 | 0 | 0 | 3  (33.3) | 0 | 0 | 0 | 5  (17.2) | 0 |
| Hirsutism^a^ | 1  (14.3) | 1  (14.3) | 0 | 0 | 1  (20.0) | 1  (20.0) | 0 | 0 | 2  (16.7) | 2  (16.7) |
| Arthralgia | 2  (10.0) | 0 | 0 | 0 | 2  (22.2) | 0 | 0 | 0 | 4  (13.8) | 0 |
| Chills | 2  (10.0) | 0 | 0 | 0 | 2  (22.2) | 0 | 0 | 0 | 4  (13.8) | 0 |
| Depression | 1  (5.0) | 0 | 0 | 0 | 3  (33.3) | 0 | 0 | 0 | 4  (13.8) | 0 |
| Dizziness | 1  (5.0) | 0 | 0 | 0 | 3  (33.3) | 0 | 0 | 0 | 4  (13.8) | 0 |
| Dysgeusia | 3  (15.0) | 2  (10.0) | 0 | 0 | 1  (11.1) | 0 | 0 | 0 | 4  (13.8) | 2  (6.9) |
| Gamma-glutamyltransferase increased | 3  (15.0) | 0 | 3  (15.0) | 0 | 1  (11.1) | 0 | 1  (11.1) | 0 | 4  (13.8) | 0 |
| Muscle spasms | 0 | 0 | 0 | 0 | 4  (44.4) | 2  (22.2) | 1  (11.1) | 0 | 4  (13.8) | 2  (6.9) |
| Musculoskeletal pain | 1  (5.0) | 0 | 0 | 0 | 3  (33.3) | 0 | 1  (11.1) | 0 | 4  (13.8) | 0 |
| Pain in extremity | 1  (5.0) | 1  (5.0) | 0 | 0 | 3  (33.3) | 0 | 0 | 0 | 4  (13.8) | 1  (3.4) |
| Weight decreased | 2  (10.0) | 0 | 0 | 0 | 2  (22.2) | 0 | 0 | 0 | 4  (13.8) | 0 |
| Anemia | 3  (15.0) | 0 | 1  (5.0) | 0 | 0 | 0 | 0 | 0 | 3  (10.3) | 0 |
| Blood alkaline phosphatase increased | 3  (15.0) | 0 | 3  (15.0) | 0 | 0 | 0 | 0 | 0 | 3  (10.3) | 0 |
| Constipation | 2  (10.0) | 0 | 0 | 0 | 1  (11.1) | 0 | 0 | 0 | 3  (10.3) | 0 |
| Dysphonia | 1  (5.0) | 0 | 0 | 0 | 2  (22.2) | 0 | 0 | 0 | 3  (10.3) | 0 |
| Hypoalbuminemia | 3  (15.0) | 0 | 0 | 0 | 0 | 0 | 0 | 0 | 3  (10.3) | 0 |
| Hypocalcemia | 3  (15.0) | 1  (5.0) | 0 | 0 | 0 | 0 | 0 | 0 | 3  (10.3) | 1  (3.4) |
| Hyponatremia | 3  (15.0) | 0 | 1  (5.0) | 0 | 0 | 0 | 0 | 0 | 3  (10.3) | 0 |
| Hypotension | 1  (5.0) | 0 | 1  (5.0) | 0 | 2  (22.2) | 1  (11.1) | 0 | 0 | 3  (10.3) | 1  (3.4) |
| Muscular weakness | 2  (10.0) | 0 | 1  (5.0) | 0 | 1  (11.1) | 0 | 0 | 0 | 3  (10.3) | 0 |
| Productive cough | 2  (10.0) | 0 | 0 | 0 | 1  (11.1) | 0 | 0 | 0 | 3  (10.3) | 0 |
| Vomiting | 1  (5.0) | 0 | 0 | 0 | 2  (22.2) | 0 | 0 | 0 | 3  (10.3) | 0 |

*Coh* cohort, *MedDRA* Medical Dictionary for Regulatory Activities, *n* number of patients with at least 1 TEAE, *N* number of patients in population, *Sch* schedule, *TEAE* treatment-emergent adverse event

^a^ Denominator adjusted because gender-specific event for females: N=2 (Sch1 Coh1), N=0 (Sch1 Coh2), N=3 (Sch1 Coh3), N=2 (Sch1 Coh4), N=2 (Sch2 Coh1), N=3 (Sch2 Coh2)
